# A six-week longitudinal dataset of wearable and self-reported stress measurements in working adults

**DOI:** 10.1038/s41597-026-07711-4

**Published:** 2026-07-02

**Authors:** Christina Halmich, Oliver Jung, Mathias Schmoigl-Tonis, Christoph Schranz, Wolfgang Kremser, Beatrix Kunas, Anton Laireiter

**Affiliations:** 1 Salzburg Research Forschungsgesellschaft mbH, Human Motion Analytics, Salzburg, 5020 Austria; 2 Paris-Lodron University Salzburg, Department of Artificial Intelligence and Human Interfaces, Salzburg, 5020 Austria; 3 Paris-Lodron University Salzburg, Department of Psychology, Salzburg, 5020 Austria

## Abstract

Stress plays a significant role in energy regulation and mental health. However, the dynamics of long-term stress patterns in daily life and the influence of stress valence on chronic stress and psychological health remain insufficiently understood. Wearable sensors offer an unobtrusive way to measure individual stress responses in daily life by enabling continuous, long-term recording of physiological signals in naturalistic settings, thereby providing a quantitative basis for understanding and assessing stress and its valence over time. To this end, we present a dataset collected from 31 participants in a six-week longitudinal study of stress conducted in a naturalistic setting. Data were acquired using the RELAX mobile-health app that provides just-in-time adaptive interventions (JITAIs) to support occupational stress management. The dataset includes both objective measurements acquired from wearable sensors and subjective self-reported stress assessments. Physiological measures comprise continuously recorded interbeat intervals (IBIs) and accelerometer (ACC) signals sampled at 52 Hz, while subjective stress data were assessed multiple times per day in addition to morning and evening questionnaires.

## Background & Summary

Occupational stress is widely recognized for its negative impact on employees’ mental and physical health, as well as its economic burden on organizations^1–4^. Traditional approaches to workplace stress management often rely on generalized interventions and therefore fail to account for substantial inter-individual differences in stress perception and response^5,6^. Prior work has highlighted the importance of distinguishing between positively perceived stress (*eustress*) and negatively perceived stress (*distress*), as stress valence may differentially relate to long-term physiological health^7^. However, empirical investigation of stress valence in daily life remains limited due to the scarcity of longitudinal, multimodal datasets combining physiological measurements with frequent self-reported assessments in naturalistic occupational settings.

To address this gap, we developed the RELAX stress management app, i.e. *RelaxApp*, as a platform for longitudinal stress data collection in everyday working environments. It was custom-built as an in-house research application for the present study^8^. The app remains proprietary and is available only as part of institutional research or service activities. *RelaxApp* integrates subjective self-reports with continuous physiological measurements acquired from *Polar Verity Sense* wearable sensors, enabling the capture of stress-related dynamics over extended periods^9,10^. In addition to data collection, the application delivers just-in-time adaptive interventions (JITAIs)^11^, whose timing and content are adjusted based on user-reported and sensor-derived indicators^12,13^. These JITAIs are brief microinterventions for occupational stress management. The intervention pool included approaches derived from cognitive behavioral therapy and health psychology, including psychoeducation, skills and attitude training, relaxation exercises, and health-habit-related prompts. Interventions were designed to be completed within a short time period so that they could be integrated into everyday working life. Depending on the intervention type, content was presented as text, tables, task prompts, assisted app-guided exercises, open self-reflection prompts, pseudochat-based guidance, audio, or video material. In the app, interventions were selected through a decision-tree procedure based on the user’s current stress context, with later personalization based on user feedback ratings. A detailed description of the *RelaxApp* intervention logic, intervention categories, presentation formats, and personalization procedure is provided in^14^.

**Fig. 1:**
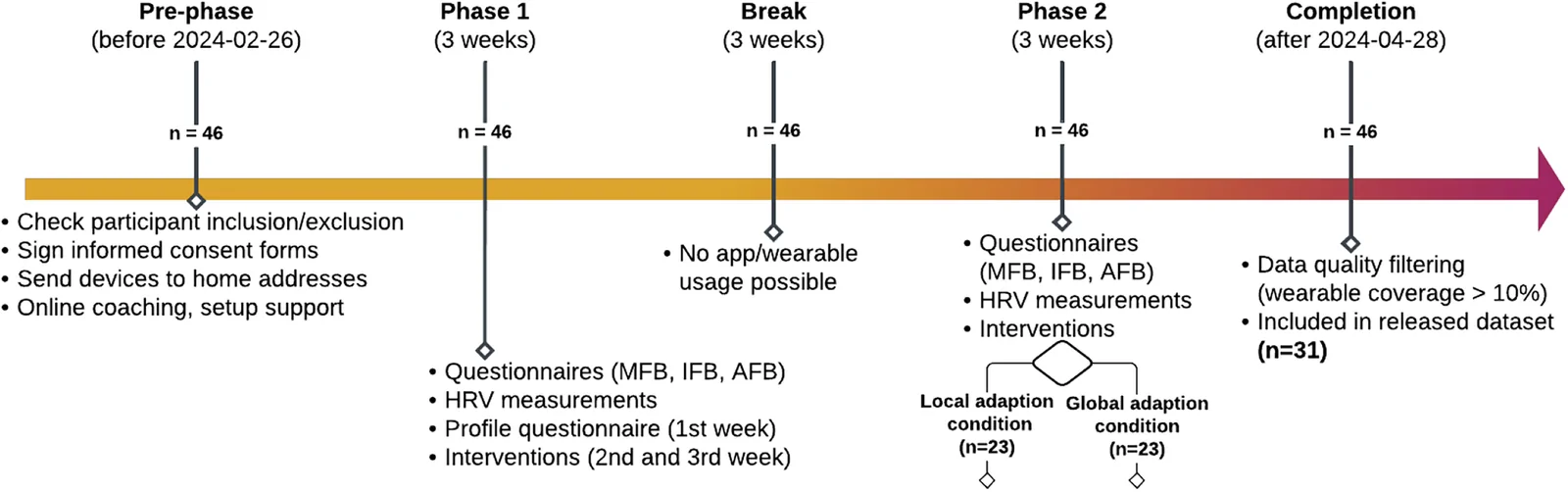
Timeline: Overview of study phases, participant flow, intervention allocation, and data inclusion in the six-week RELAX longitudinal field study. Abbreviations: morning questionnaire (MFB), interval questionnaire (IFB), and evening questionnaire (AFB).

This Data Descriptor introduces the dataset collected during the evaluation study of the *RelaxApp* (Fig. 1). It provides comprehensive documentation and public access to the full dataset to support independent validation, secondary analyses, and methodological development.

The study comprised two consecutive three-week phases separated by a break. During the first phase, all participants used the *RelaxApp* to record subjective stress assessments and wearable-derived physiological signals. In the second phase, participants were randomly assigned to one of two intervention strategies.

The dataset has been used in previous analyses conducted by our research group and collaborators. These publications investigated topics including stress detection from wearable signals, multimodal modeling of physiological and self-reported stress dynamics, and the evaluation of adaptive intervention strategies^7,8,12,14–16^.

Several publicly available datasets have contributed to research on wearable-based stress and affect detection. The WESAD dataset^17^ provides multimodal physiological recordings collected under controlled laboratory stress induction, including chest- and wrist-worn sensors during predefined affective tasks. The LifeSnaps dataset^18^ offers longitudinal smartwatch data combined with ecological self-reports, enabling investigation of real-world behavioral and physiological patterns.

In contrast to laboratory-based datasets, the present dataset captures wearable-derived physiological measurements and repeated self-reported stress assessments over a six-week period in a naturalistic occupational context. The dataset can be used to investigate longitudinal associations between wearable-derived physiological signals and self-reported stress in naturalistic work settings. It further enables comparative analyses of stress-related physiological and subjective dynamics under different levels of intervention personalization, as well as the development and validation of algorithms for stress detection, activity recognition, and multimodal time series modeling.

## Methods

### Ethical approval

The study described in this paper was approved by the ethics committee of the University of Salzburg (vote number EK-GZ 25/2023) and was conducted following the ethical standards of the 1964 Declaration of Helsinki, including its amendments. All participants provided written informed consent prior to participation. The participant information and consent form explicitly informed participants that study data would be processed in anonymized or pseudonymized form, that direct identifiers would be stored separately from study data, and that results and data would only be communicated to other researchers or the public in anonymized form. Participants were also informed that anonymized data may be used, where ethically and legally permissible, for further scientific purposes, including open-science databases, new types of analyses, and additional publications. The public dataset released with this Data Descriptor contains no direct personal identifiers and was prepared for dissemination in accordance with the approved consent procedure and ethics approval. To further reduce re-identification risk in the public release, indirect identifiers were minimized before publication. The exact year-of-birth information was replaced by 10-year birth-year brackets, and personal free-text variables, including the open-text hobby variable, were removed from the public dataset.

### Study design

The dataset was collected as part of a longitudinal field study designed to capture stress-related physiological and subjective data in a naturalistic occupational setting over an extended period. The study followed a two-phase design spanning six weeks, with continuous data collection throughout both three-week phases separated by a break period, as depicted in Fig. 1. During the first phase (starting 2024-02-26), all participants used a mobile-health application called *RelaxApp* on their personal smartphones to record subjective stress assessments, while continuous physiological data were collected via a wearable sensor worn on the upper arm. This phase served to establish baseline stress-related patterns under a uniform intervention strategy. Interventions were introduced from the second week of Phase 1 onward and were applied uniformly across participants during the remainder of this phase, while physiological and questionnaire-based measurements continued throughout.

In the second phase (starting 2024-04-08), participants continued to use the *RelaxApp* and were randomly assigned to one of two intervention adaptation strategies. In the global adaptation condition, intervention adjustments were informed by aggregated feedback derived from all study participants. In the local adaptation condition, intervention personalization placed greater weight on data and preferences from the individual user.

Specifically, intervention-selection weights were updated based on user feedback ratings. In the global adaptation component, the selection weight of an intervention was multiplied by a factor of ${\sqrt{2}}^{{n}_{{\rm{inter}}-{\rm{sum}}}/{n}_{{\rm{subjects}}}}$, where ${n}_{{\rm{inter}}-{\rm{sum}}}$ denotes the summed feedback score for that intervention across users and ${n}_{{\rm{subjects}}}$ the number of contributing participants. Thus, an intervention with an average summed positive feedback score of 2 per participant would have its selection weight doubled, whereas an average summed negative feedback score of −2 would halve its selection weight. In the local adaptation condition, the same weighting principle was additionally applied using only feedback from the individual user, with ${n}_{{\rm{subjects}}}\mathrm{=1}$.

Apart from the adaptation strategy, all other aspects of data collection and application functionality remained unchanged between groups. Continuous physiological monitoring and repeated self-reported stress assessments were maintained throughout Phase 2 in both intervention conditions.

Throughout the study, the *RelaxApp* prompted participants to complete repeated self-reported stress assessments during the day, as well as standardized questionnaires in the morning and evening. In parallel, physiological signals, including interbeat intervals (IBIs) and accelerometer (ACC) data, were continuously recorded by the wearable device and transmitted to the RELAX backend infrastructure for storage and processing.

### Participant recruitment

Study participants were recruited between December 2023 and January 2024 through institutional mailing lists and internal communication channels at partnering organizations such as social media, the study website, or flyers.

Eligible participants were of legal age and employed with regular working hours, not interrupted by holidays or significant changes in employment status, or travel during the study period. Participants were required to be fluent in the German language and have corrected-to-normal eyesight. Participants were required to own a smartphone running at least Android version 7.0 and to have sufficient availability to engage with the mobile application on a daily basis.

Excluded were participants with irregular working hours (e.g. night shifts), a history of cardiovascular diseases, atypical menstrual cycle disturbances or upcoming medical interventions during the study period. Ongoing psychotherapy or psychopharmacological treatment, as well as diagnosed severe mental disorder, also led to exclusion.

Eligibility criteria were assessed primarily via self-report unless circumstances allowed direct clarification. No limitations were imposed regarding participants’ occupational sectors or their residence within German-speaking countries. Communication with the research team was conducted remotely via email or telephone.

A total of 50 participants were enrolled in the study and completed the initial onboarding procedure. During the course of the study, four participants discontinued participation, resulting in 46 participants completing both study phases. However, usable wearable-derived physiological data were not available for all completers.

For 15 participants, physiological recordings were incomplete or unusable because of technical recording failures, smartphone or operating-system updates during the study period, Bluetooth/connectivity problems, or extended periods of sensor non-wear/non-compliance that were not always detectable during ongoing data collection. The released dataset therefore includes the 31 participants for whom both questionnaire-based self-reports and wearable-derived physiological time series were available after applying the predefined data-quality and completeness criteria. Inclusion required at least 10% overall physiological recording coverage across the total measurement duration on a 24-hour basis. Participants below this threshold were not included because their physiological time series were considered insufficient for meaningful reuse or validation of longitudinal wearable-derived stress measures.

### Dataset

The dataset is a multimodal longitudinal dataset designed to capture stress-related dynamics in a naturalistic occupational setting, combining continuously recorded physiological signals from wearable sensors with frequent subjective self-reports collected via a mobile application over two three-week study periods. All data were collected in participants’ everyday working environments without laboratory constraints, providing measurements of stress-related processes. The dataset is intended to support methodological research on stress modeling, validation of wearable-based stress indicators, and the development of personalized stress monitoring approaches.

#### Wearable measurements

Physiological data were acquired using a *Polar Verity Sense* wearable sensor (*Polar Electro Oy, Finland*) worn on the upper arm. The sensor was accessed using the Polar BLE SDK version 5.1.0 and firmware version 2.1.0. These data include photoplethysmography (PPG) measurements as well as triaxial accelerometer signals capturing movement and activity patterns. According to the manufacturer documentation, the accelerometer supports selectable measurement ranges of ±2 g, ±4 g, ±8 g, and ±16 g with 16-bit resolution^19^. The accelerometer axes represent the device-relative coordinate system of the sensor rather than fixed anatomical axes. Polar provides an axis-orientation diagram for the *Polar Verity Sense* in the public Polar BLE SDK GitHub issue tracker^20^.

Out of the box, *Polar Verity Sense* features the on-board extraction of IBI data (referred to by the manufacturer as pulse-to-pulse intervals (PPI)). These intervals represent the time elapsed between successive cardiac pulses. In the present study, the wearable sensor was paired with the Android-based *RelaxApp*.

Physiological data were recorded during wear periods and temporarily buffered on the internal memory of the Polar Verity Sense device. The *RelaxApp* retrieved buffered data at regular intervals via Bluetooth Low Energy by initiating bulk transfers of the records stored on the device. Transfer completeness was verified using checksum-based validation. After successful transfer, the retrieved data were stored locally on the Android smartphone and removed from the sensor memory. When a stable internet connection was available, a separate background process forwarded the locally stored data to the study backend via a REST interface, storing them in a time series database (InfluxDB), again using checksum-based validation before deleting the local smartphone copy. This two-stage transfer procedure was designed to reduce data loss due to intermittent Bluetooth or internet connectivity and to avoid duplicate records. Because the Polar device cannot simultaneously write new records to firmware memory during bulk transfer, short gaps of a few seconds may occur at transfer times.

#### Daily questionnaires

In parallel, subjective stress assessments were collected repeatedly throughout the day and complemented by standardized questionnaires administered in the morning and evening. Questionnaire items were assessed using Likert-type response scales ranging from 4 to 7 points, with construct-specific semantic anchors (e.g., agreement, frequency, intensity, or valence), as detailed in the accompanying metadata file. All questionnaires were administered in German. English translations of all items and response scales are available in the metadata spreadsheet. The combination of objective physiological measurements and subjective self-reports enables analyses of stress perception, stress valence, and their association with wearable-derived physiological responses in daily life. The following questionnaires were administered daily:

- **Morning questionnaire (MFB):** The *Morgen-Fragebogen (MFB)* assessed participants’ subjective state at the beginning of the day, including (a) current valence, (b) arousal, (c) expected stress for the day, and (d) subjective sleep quality of the preceding night.
- **Evening questionnaire (AFB):** The *Abend-Fragebogen (AFB)* captured retrospective evaluations of the day, including (a) valence during the day, (b) arousal during the day, (c) well-being during the day, and (d) chronic subjective stress.
- **Interval questionnaire (IFB):** Push notifications prompting completion of the *Intervall-Fragebogen (IFB)* were sent two to three randomized times per day during working hours. Participants could choose to complete it following the notification and were also able to initiate it voluntarily. The IFB assessed momentary (a) valence, (b) arousal, as well as (c) perceived valence and (d) arousal of the most recent stressful event.

Together, these instruments provide dense longitudinal self-report data that complement the continuous physiological measurements.

#### Participant profile

During the first week of the study, participants completed a set of questionnaires to characterize their individual profiles and demographic composition. These questionnaires assessed living circumstances, workplace conditions, technology competence, everyday and work-related stressors, general risk factors, available resources, and coping strategies related to stress. The collected information provides contextual variables that can be used to stratify participants or to control for inter-individual differences in subsequent analyses.

## Data Records

### Availability of data files

The proposed dataset is **publicly available at** https://doi.org/10.5281/zenodo.18693288^21^ and comprises wearable-derived physiological measurements and questionnaire-based self-reported data collected during a six-week longitudinal field study. Although both data types were collected within the same study period, they differ substantially in their temporal characteristics and data representation. Questionnaire responses represent discrete events associated with specific completion times, whereas wearable measurements constitute continuous time series recordings.

**Fig. 2:**
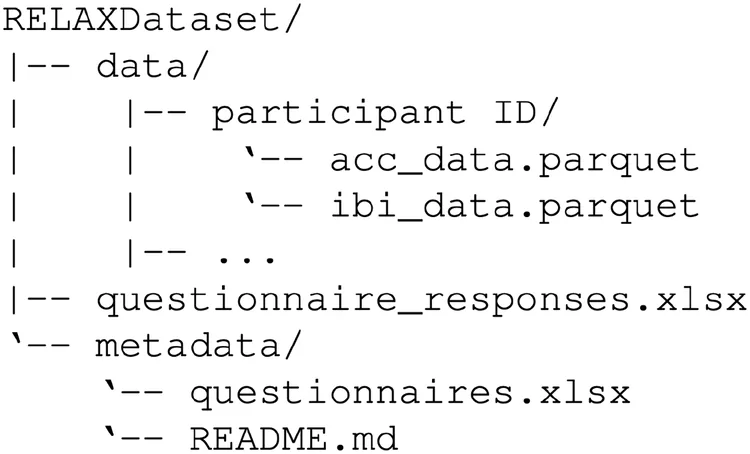
File structure of the dataset as released on Zenodo.

The dataset is provided as a directory-based file structure (Fig. 2). Data are organized participant-wise and temporal relationships are encoded explicitly within the data files using timestamp columns.

This design prioritizes efficient storage, scalable analysis of large time series data, and flexibility in downstream processing, while preserving full temporal and participant-level traceability.

### Data overview

The released dataset consists of two main data components: questionnaire-based self-reported data collected through the *RelaxApp* and wearable-derived physiological time series data recorded with the *Polar Verity Sense*. The following sections describe the structure, content, and file formats of each data component.

#### Self-reported data

Questionnaire-based self-reported data are provided in spreadsheet format in their original German language and include morning, evening, and interval questionnaires. Each questionnaire response is associated with a pseudonymized participant identifier and recorded timestamp information, enabling temporal alignment with the physiological measurements. Questionnaire metadata, including question texts, response scales, and answer options, are provided in a dedicated metadata spreadsheet in their original language and English translations. English translations are intended for documentation purposes only and have not undergone formal psychometric validation.

Table 1 shows the demographic characterization of the released cohort, including gender, age, BMI, education level, and occupational sector of the participants.

**Table 1:** Demographic characterization of the released cohort.

	N	%	Mean	STD	Min	Max
**Gender**	**31**	**100**				
female	20	64.5				
male	11	35.5				
**Age**			40	9.34	25	63
**BMI**			25.15	4.65	19.61	39.68
**Education Level**						
Postgraduate university degree/Master’s	11	35.5				
Graduate university degree/Bachelor	7	22.6				
Abitur/Fachabitur/Matura	6	19.4				
Apprenticeship/technical school	4	12.9				
Higher (from doctorate)	2	6.5				
Compulsory school	1	3.2				
Occupational Sector*						
Services	10					
Society, Sport and Culture	5					
Trade	4					
Energy and Environment	3					
Finance, Insurance and Real Estate	2					
Health	2					
Industry	2					
Media	1					
Chemistry, Metal and Raw Materials	1					
Tourism, Gastronomy and Leisure	1					
Politics	1					

*For occupational sector, multiple answers can be selected.

#### Wearable data

The wearable component of the released dataset comprises time series data from 31 participants. For each participant, one ACC and one IBI file are provided. Across both modalities, six primary features are included. Wearable-derived physiological data are provided as modality-specific Apache Parquet files, an open, columnar file format suitable for efficient storage and access to large time series datasets.

Accelerometer (ACC) data contains three triaxial acceleration signals (‘acc_x’, ‘acc_y’, ‘acc_z’) as floating-point values and a ‘timestamp’ column stored as UTC datetime objects. Accelerometer measurements were sampled at approximately 52 Hz, corresponding to a median inter-sample interval of approximately 19.23 ms.

Interbeat Interval (IBI) data are event-based, with each record representing the temporal duration between successive heartbeat measurements as PPIs. IBI data contains three event-based features ‘ibi_ppi’ (integer, milliseconds), ‘ibi_errorEstimate’ (integer, milliseconds), ‘ibi_blocker’ (boolean) and a ‘timestamp’ column stored as UTC datetime objects. Each heartbeat event ‘ibi_ppi’ is accompanied by an ‘ibi_errorEstimate’ value, which provides a sensor-specific estimate of the temporal accuracy. In addition, ‘ibi_blocker’ indicates issues in data quality, by marking intervals during which reliable detection was temporarily suspended. In contrast to the fixed-rate ACC data, IBI measurements are irregularly spaced in time, reflecting physiological heart rhythm dynamics. These structural characteristics formed the basis for subsequent validation procedures, including sampling-rate verification, timestamp integrity checks, completeness assessment, and physiological plausibility analysis.

## Technical Validation

The dataset underwent systematic validation to ensure structural integrity, completeness, and consistency of the wearable measurements. Validation procedures included checking that each data file contained the expected variables, column names and data types, confirming that files could be opened and parsed successfully, identifying empty files or records with missing, incomplete, or non-numeric values where numeric measurements were expected, and assessing data availability at the modality and study-phase levels.

The released dataset includes wearable recordings from 31 participants. For each participant, one accelerometer (‘acc_data’) file and one interbeat interval (‘ibi_data’) file are provided. All expected modality files were present. No participant-level ACC or IBI files were missing in the published dataset.

### Phase-level data availability

Although all processed files were structurally valid, phase-level data absences were present in the original raw data for a small number of participants. Specifically:

- Participant 27: No ACC or IBI data available between 2024-04-21T22:00:00.000Z and 2024-04-28T21:59:59.999Z.
- Participant 57: No IBI data available between 2024-03-03T23:00:00.000Z and 2024-03-17T22:59:59.999Z.
- Participant 63: No ACC or IBI data available between 2024-02-25T23:00:00.000Z and 2024-03-03T22:59:59.999Z, and between 2024-04-21T22:00:00.000Z and 2024-04-28T21:59:59.999Z.

These absences correspond to complete modality-interval combinations that were not recorded in the original dataset. No additional data loss occurred during preprocessing.

During preprocessing, weekly files were programmatically aggregated into per-participant ACC and IBI time series files. Missing weeks were detected automatically and skipped without introducing artificial data. As a result, the released dataset faithfully reflects the availability of the original recordings. No imputation, interpolation, or reconstruction of missing phases was performed. All wearable data files were successfully parsed after preprocessing. No corrupted, unreadable, or structurally malformed files were detected.

### Timestamp integrity

Timestamp integrity was verified separately for ACC and IBI data. During preprocessing, raw files were concatenated into a single continuous time series per participant. After concatenation, timestamps were confirmed to be strictly increasing, with no duplicate entries or temporal overlaps detected within any file. No backward time shifts or timestamp resets were observed. All timestamps fell within the predefined study period and corresponding phase boundaries.

The empirical accelerometer sampling frequency was 52 Hz across participant-days, confirming stable fixed-rate acquisition without observable timing drift.

For IBI data, timestamps exhibited irregular spacing as expected for event-based heartbeat detection. Very small deviations (<0.2 ms) were attributable to floating-point precision effects rather than true temporal inversions.

Larger inter-event gaps indicate interruptions in valid heartbeat detection and may reflect non-wear, temporary sensor detachment, or insufficient signal quality e.g. due to severe motion artefacts. In subsequent availability analyses, these intervals were treated as missing IBI coverage rather than valid physiological observations.

### IBI interval validation

All IBIs (‘ibi_ppi’) were required to be strictly positive. Using a conservative physiological plausibility range of 300–2000 ms, approximately 1.19% of intervals fell outside this range. These extreme values are consistent with occasional motion artifacts or missed detections in long-term PPG-based sensing and were retained in the released dataset to preserve raw signal characteristics.

After restricting to physiologically plausible intervals (300–2000 ms), the global median IBI was 846 ms (IQR: 726–986 ms), corresponding to typical resting-to-moderate heart rate ranges in adults. The 95th percentile of plausible intervals was 1379 ms, further supporting physiological consistency.

The device-reported ‘ibi_errorEstimate’ had a median of 15 ms (IQR: 11–30 ms), indicating generally acceptable temporal precision of heartbeat detection under field conditions. The 95th percentile of the error estimate was 490 ms, reflecting occasional intervals with elevated uncertainty.

The motion-related quality flag (‘ibi_blocker’) was active for 35.95% of intervals, indicating that a substantial proportion of heartbeat detections occurred under movement conditions in this naturalistic field study. These quality indicators are retained to enable application-specific artifact filtering and signal processing.

### Data completeness and consistency

Tables 2 and 3 summarize data availability for ACC and IBI recordings across all participants, aggregated by week. Reported values represent the mean proportion of recorded data relative to 24 hours per day and the corresponding mean daily recording duration in hours (±standard deviation).

**Table 2:** Proportion of available acceleration data and corresponding daily average durations, aggregated across all subjects for each week, with standard deviations indicating variability among participants.

Week number	Proportion of available ACC data (%, mean ± std)	Daily data (hours) (mean ± std)
Week 1	52.05 ± 15.67	12.83 ± 3.59
Week 2	46.77 ± 15.58	11.29 ± 3.70
Week 3	35.85 ± 14.50	9.25 ± 3.05
Week 4	30.17 ± 15.89	8.14 ± 3.46
Week 5	33.36 ± 15.27	8.56 ± 3.36
Week 6	27.56 ± 16.65	7.56 ± 3.59
**Total**	37.59 ± 17.75	9.60 ± 3.89

**Table 3:** Proportion of available interbeat interval data and corresponding daily average durations, aggregated across all subjects for each week, with standard deviations indicating variability among participants.

Week number	Proportion of available IBI data (%, mean ± std)	Daily data (hours) (mean ± std)
Week 1	50.54 ± 14.27	12.42 ± 3.29
Week 2	45.82 ± 14.72	11.06 ± 3.47
Week 3	35.07 ± 14.28	9.06 ± 3.04
Week 4	30.16 ± 15.44	7.99 ± 3.31
Week 5	33.14 ± 14.46	8.54 ± 3.09
Week 6	26.81 ± 16.54	7.37 ± 3.63
**Total**	36.89 ± 17.02	9.40 ± 3.71

For ACC data, the mean daily coverage decreased from $52.05 \% (12.83\pm 3.59h)$ in Week 1 to $27.56 \% (7.56\pm 3.59h)$ in Week 6. A comparable pattern was observed for IBI data, with mean daily coverage decreasing from $50.54 \% (12.42\pm 3.29h)$ in Week 1 to $26.81 \% (7.37\pm 3.63h)$ in Week 6. Across the entire six-week period, overall mean coverage amounted to $37.59 \% (9.60\pm 3.89h)$ for ACC and $36.89 \% (9.40\pm 3.71h)$ for IBI recordings. Across all participants and study days, the median daily coverage was $41.3 \% (IQR:24.2-56.7 \% )$ for accelerometer recordings and $40.0 \% (IQR:23.2-56.4 \% )$ for interbeat interval recordings.

As both signals originate from the same wearable device, coverage estimates derived from ACC and IBI modalities were closely aligned, primarily reflecting device wear-time with minor deviations attributable to the event-based nature of IBI detection and temporary motion-related suppression of heartbeat interval extraction. The relatively large standard deviations across weeks indicate substantial inter-individual variability in wearable data availability. Variability increased in later weeks, reflecting greater heterogeneity in recording duration across participants, with some participants maintaining consistently high daily coverage and others exhibiting frequent partial or complete non-wear days.

**Fig. 3:**
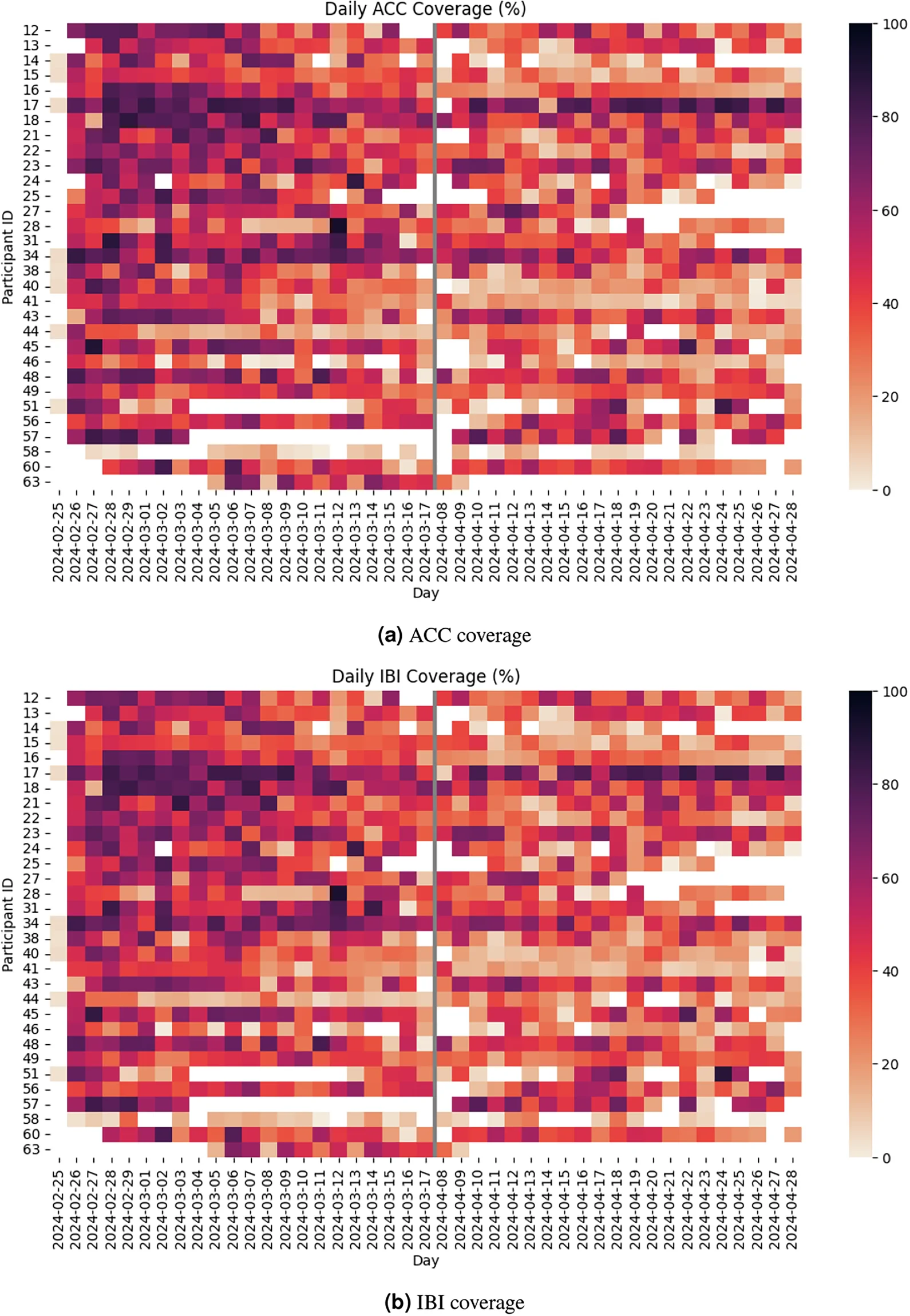
Heatmaps of daily data coverage per participant across the study period for accelerometer (ACC) and interbeat interval (IBI) data. Each row represents one participant and each column one calendar day. Color intensity indicates the proportion of recorded data relative to 24 hours, with lighter colors reflecting lower coverage and white cells indicating days without recorded data. The vertical gray line marks the study pause period.

The temporal structure of this variability for ACC and IBI data is illustrated in Fig. 3. Both show highly comparable temporal coverage patterns. The heatmap shows daily coverage for each participant across the six-week study period. Each row represents one participant and each column one calendar day. Color intensity reflects the proportion of recorded data relative to 24 hours, with lighter colors indicating lower coverage and white cells indicating days without recorded data. The heatmap reveals substantial inter-individual variability in daily coverage. While several participants exhibit consistently moderate to high daily coverage throughout the study, others show intermittent or prolonged periods of low coverage or complete non-wear. Temporal clustering of missing days is observable in later study weeks for multiple participants, consistent with the overall decline in mean weekly coverage reported in Table 2. Coverage patterns appear largely participant-specific rather than synchronized across subjects, indicating that missingness predominantly varies at the participant level.

**Fig. 4:**
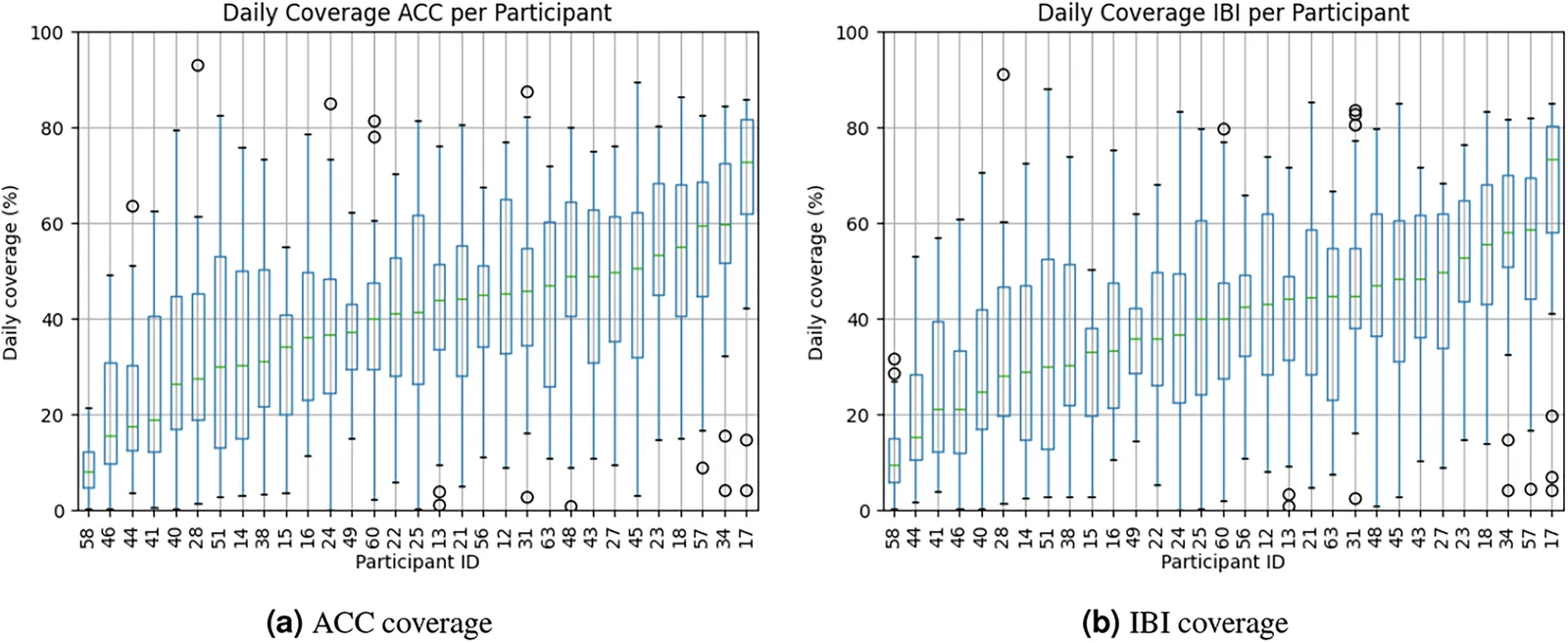
Distribution of daily data coverage (%) per participant across the six-week study period for ACC and IBI data. Boxplots represent the median (central line), interquartile range (box), and range excluding outliers (whiskers); points indicate outlier days. Substantial inter-individual variability in data availability is observable across participants.

While Fig. 3 illustrates temporal patterns of missingness, Fig. 4 summarizes the distribution of daily coverage per participant across the study period. It presents the distribution of daily ACC and IBI coverage per participant. Boxplots display the median, interquartile range, and range excluding outliers. The figure further emphasizes substantial inter-individual variability, with median daily coverage ranging from below 20% to above 60% across participants.

## Usage Notes

Wearable-derived physiological data are provided in Apache Parquet format, while questionnaire responses and metadata are provided in spreadsheet format. These open, non-proprietary formats can be accessed using standard data analysis libraries in common programming environments (e.g., Python or R), and no proprietary software is required. For instance, in Python, the ‘fastparquet’^22^ library enables efficient reading and writing of Parquet files. In R, the ‘arrow’ package^23^ provides functions to handle Parquet data.

The data were collected under naturalistic, real-world conditions. As a result, wearable data availability varies across participants and over time, and missing data are present. No imputation has been applied to the released dataset. Researchers are encouraged to explicitly assess data coverage and to apply preprocessing and missing-data handling strategies appropriate to their specific research questions.

Accelerometer data are sampled at approximately 52 Hz and represent fixed-rate time series. In contrast, interbeat interval (IBI) data are event-based and irregularly spaced. Analyses requiring uniformly sampled signals should apply appropriate interpolation or resampling procedures prior to feature extraction. Device-reported quality indicators (‘ibi_errorEstimate’ and ‘ibi_blocker’) are included and may be used for artifact detection or signal quality filtering depending on the intended application.

Several limitations should be considered when reusing the dataset. Although participants were recruited through multiple channels, the sample may not be fully representative of the general working population. In particular, individuals with an affinity for technology-assisted stress management may be overrepresented, introducing potential unit non-response bias. In addition, missing wearable data, often resulting from participants not wearing the sensor, may exhibit non-random patterns. For example, if participants preferentially removed the device during rest or recovery periods, physiological patterns associated with regeneration may be underrepresented in the continuous recordings. These characteristics should be taken into account when interpreting results derived from the dataset.

## Data Availability

The dataset described in this Data Descriptor is publicly available at Zenodo under https://doi.org/10.5281/zenodo.18693288. The repository contains accelerometer (ACC) and interbeat interval (IBI) recordings for each participant in Apache Parquet format, questionnaire responses in spreadsheet format, and accompanying metadata and documentation files. All data are fully pseudonymized and provided in open, non-proprietary formats to support transparent and reproducible reuse.

## Code availability

The custom validation code used to generate the completeness, coverage, timestamp integrity, and physiological plausibility checks reported in the Technical Validation section is publicly available at https://github.com/halmichchristina/longitudinal-stress-dataset/^24^. Custom code was also used for data acquisition, backend communication, data export, and preprocessing. The data acquisition code of the *RelaxApp*, including the streaming backend and related services, is not publicly released because the application was custom-built as a proprietary in-house research application under further development. The overall RELAX system concept and technical framework have been described previously^8^.
